# Radioactive Merano SPA Treatment for Allergic Rhinitis Therapy

**DOI:** 10.1155/2016/2801913

**Published:** 2016-09-08

**Authors:** Desiderio Passali, Giacomo Gabelli, Giulio Cesare Passali, Roberto Magnato, Stefan Platzgummer, Lorenzo Salerni, Salvatore Lo Cunsolo, Alexandra Joos, Luisa Maria Bellussi

**Affiliations:** ^1^ENT Department, University of Siena, Siena, Italy; ^2^ENT Department, Sacred Heart University, Roma, Italy; ^3^ENT Department, Tappeiner Hospital, Merano, Italy; ^4^Clinical Chemistry and Microbiology Laboratory, Tappeiner Hospital, Merano, Italy; ^5^Health Unit, Terme Merano, Merano, Italy

## Abstract

Allergic rhinitis is a common nasal disorder with a high impact on quality of life, high social costs in therapies, and a natural development towards asthma. Pharmacological therapy is based on several genres of medications, of which intranasal corticosteroids are currently the most widespread. Thermal water treatment has traditionally been used as adjunctive treatment for chronic rhinitis and sinusitis. The present study was carried out to assess the clinical efficacy of nasal inhalation of radioactive oligomineral water vapours from the Merano hot spring and to compare it with the clinical efficacy of mometasone furoate nasal spray. A comparative prospective study was performed in 90 allergic patients treated at Merano hot springs: a group of 54 subjects treated with radioactive thermal oligomineral water and a control group of 36 subjects treated with mometasone nasal spray. Patients of both groups were assessed before and after treatment by Sino-Nasal Outcome Test questionnaire, active anterior rhinomanometry with flow and resistance monitoring, measurement of mucociliary transport time, and cytological examination of nasal brushing/scraping. The study showed that inhalation treatment with radioactive hydrofluoric thermal water for two weeks produces an objective clinical and cytological improvement in allergic patients, similar to that obtained with mometasone furoate nasal spray.

## 1. Introduction

Allergic rhinitis is a common chronic nasal disorder with a high impact on quality of life, high social costs in therapies, and a natural development towards asthma. Its pathogenesis is based on an immune reaction against inhaled allergens. Sensitization to allergens is regulated by Th2 cells. When a person is exposed to allergens, to which he/she is sensitized, cross bonding occurs between IgE and allergens on mast cells, causing typical nasal symptoms within a few minutes. The symptoms are due to release of neuro- and vasoactive mediators, such as histamine, IL-4, leukotrienes, and prostaglandins [1]. At histological-cytological level, the normal status of nasal mucosa cells (ciliate cells, muciparous goblet cells, and basal cells) tends to change with the appearance of cells typical of inflammation, such as neutrophils, eosinophils, and mast cells.

Allergic rhinitis is defined by typical symptoms, such as nasal obstruction, abundance of clear/pale-coloured mucous secretion, itching, and sneezing. Treatment involves a combination of actions, such as limiting risk factors such as early allergen exposure, alleviating symptoms, and preventing sensitization. Pharmacological therapy is based on histamine antagonists, topical intranasal steroids, and specific immune therapy. Intranasal corticosteroids are currently the most widespread pharmacological therapy [2–4].

Thermal water treatment has traditionally been used as adjunctive treatment for chronic rhinitis, sinusitis, and bronchitis.

Several studies have found a correlation between therapy based on inhalation of mineral water with different physical and chemical characteristics (temperature, pressure, radioactivity, and the presence of specific ions or active chemical groups) and positive changes of subjective and objective parameters such as symptom and medication score, nasal resistance values, mucociliary clearance, and immunoglobulin concentration in nasal secretion and blood. They concluded that the mechanism of action of thermal treatment in chronic inflammation of the nose and paranasal sinuses is not limited to the action of cleansing, massage, and mucus dilution, but each type of thermal water has typical biological function related to its specific physical and chemical characteristics, thus implying specific indication in different pathologies [5–7].

The present study was conducted to assess the clinical efficacy of nasal inhalation of radioactive (481 Bq/l radon) oligomineral water hot vapours rich in fluorine from the Merano hot springs (Table 1) in allergy patients with nasal obstruction as main symptom. Primary outcome was the evaluation of the efficacy of the therapy by objective methods including bilateral flow and resistance measurement by active anterior rhinomanometry (AAR), mucociliary transport time (MCTt) determination, and nasal cytology. Secondary outcome was the subjective evaluation of symptoms by the Sino-Nasal Outcome Test (SNOT) questionnaire and the evaluation of treatment compliance in terms of side effects.

## 2. Methods

### 2.1. Patients

A comparative prospective study was performed with 90 allergic patients of both genders (age range 14–80 years) treated at Merano hot springs between March and October 2015. The main inclusion criterion was nasal obstruction, evaluated on the basis of medical history and a 10-Point Visual Analogue Scale, as well as a complete ENT examination. All patients enrolled in the study suffered from perennial allergic rhinitis, diagnosed by a prick test; among them 36 patients were monosensitized to D.pt. and 54 were positive to more than one allergen. They were randomly distributed in the two groups. No patient suffered from acetyl-salicylic acid intolerance, asthma, or other comorbidities. Those with acute infectious rhinitis and nasal polyps at the enrolment time were excluded. Patients who had undergone pharmacological therapy with vasoconstrictors, topical corticosteroids, NSAIDs, systemic corticosteroids, or mucolytic agents in the previous two weeks were also excluded.

The study was conducted on a group (A) of 54 subjects treated with radioactive thermal oligomineral waters and a control group (B) of 36 subjects selected by the same criteria but treated topically with mometasone furoate nasal spray for 14 days, 2 puffs/nostril every morning, each puff consisting of 50 *μ*g, equivalent to 200 *μ*g of total dose per day for each patient. The patients were allocated in the two groups randomly (Table 2).

Group A underwent 14 days of inhalation therapy with radioactive thermal oligomineral water vapours at 38°C released 20 cm from the face in 8–10 *μ*m micelles that do not penetrate beyond the upper airways. This treatment was followed by an aerosol of the same thermal water in 2–4 *μ*m micelles that reach the lower airways. Each session lasted 10 minutes.

At the time of enrolment, personal data and medical history were recorded and a complete ENT examination was performed. Patients of both groups were assessed before and after treatment by compilation of the Sino-Nasal Outcome Test (SNOT score) questionnaire that investigates subjective levels of nasal obstruction, nasal itching, rhinorrhea, sneezing, and conjunctivitis [8, 9]; active anterior rhinomanometry (AAR) with bilateral flow and resistance monitoring and measurement of mucociliary clearance time were also performed in each patient. A rhinocytogram by nasal brushing/scraping of the inferior turbinate middle third was also taken.

All the patients in the study have given their informed consent to participate; the study protocol has been approved by Tappeiner Hospital, Merano, Italy.

### 2.2. Active Anterior Rhinomanometry

Nasal ventilatory function was evaluated by active anterior rhinomanometry according to validated criteria [10], using ATMOS Rhinomanometer 300. Three to five breaths were recorded at a fixed transnasal pressure of 150 Pa, with the mouth closed and the patient in seated position. Flow expressed in cc/sec and resistance expressed in Pascal were measured for the right and left nasal fossae and as overall value. Total resistance was calculated combining the resistances of the two nostrils according to the formula

(1) $$\begin{array}{c} R_{\text{tot}}=\frac{R_{\text{left}}\times R_{\text{right}}}{R_{\text{left}}+R_{\text{right}}}. \end{array}$$

### 2.3. Mucociliary Transport

Patients with nose and sinus pathology often have low mucociliary clearance [11], assessed as an increase in mucociliary transport time, expressed in minutes. In this study mucociliary transport time (MCTt) was calculated by placing a tracer powder (charcoal) on the head of the inferior turbinate. The path of the powder was followed by direct pharyngoscopy on the posterior wall of the pharynx. The charcoal powder is an inert nonsoluble tracer which is trapped in the gel layer of the mucus and is transported passively by the movement of the cilia [12]. Transport time was calculated as the average of the values obtained from the two nasal fossae.

### 2.4. Nasal Cytology

The rhinocytogram of a healthy person shows the cells that normally constitute the ciliated pseudostratified epithelium: ciliated, muciparous, and basal cells. An increase in muciparous goblet cells and the presence of other types of cell, such as neutrophil granulocytes, eosinophils, mast cells, or fungal hyphae and bacteria, provide an indication of nasal inflammation.

In the present study, the cytological sample was obtained by bilaterally brushing or scraping the middle third of the inferior turbinate. The harvested nasal cells were placed on a microscope slide and fixed immediately for cytopathological examination. After the May-Grunwald-Giemsa staining, the slides were examined under light microscopy: examination was carried out at ×100 magnification and 6 representative microscopic fields for each slide were examined.

### 2.5. Statistical Analysis

The study was designed to compare objective clinical criteria before and after treatment. The comparison was between group A consisting of 54 subjects treated with radioactive oligomineral thermal waters and group B consisting of 36 controls selected by the same criteria but treated topically with mometasone nasal spray for 14 days.

Flow and resistance values measured by active anterior rhinomanometry (AAR) and mucociliary transport time in minutes were divided into quartiles and their statistical significance was tested by the Wilcoxon test. Differences in cell populations detected by cytological analysis were expressed as mean number of cells found on each slide for each group of patients. The statistical significance was evaluated by the Wilcoxon test.

## 3. Results

Analysis of the results was conducted including only those subjects who completed the treatment and underwent enrolment and follow-up examination.

**Figure 1:**
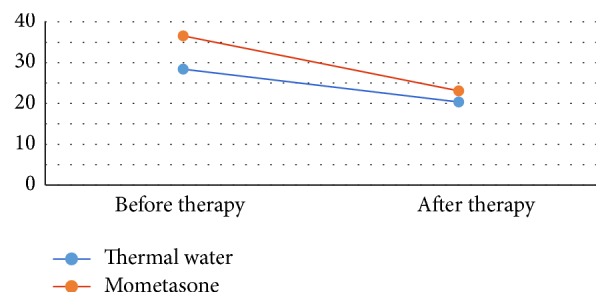
The figure shows the SNOT score value. In the patients treated with thermal water, the mean decreases from 29 points before treatment to 20 points after treatment. In the patients who follow local mometasone therapy the mean decreases as well, from 38 to 22.

The SNOT score results decreased both in the group of patients who underwent thermal water therapy, from a mean of 29 points to 20 points, and in the group following mometasone therapy, from 38 to 22 points (Table 3 and Figure 1).

With regard to combined flow and resistance data (right and left nasal fossa), the thermal treatment group showed an increase in flow from a mean of 482 cc/s to 528 cc/s (+9.54%), whereas the group treated with mometasone showed an increase in flow from a mean of 470 cc/s to 492 cc/s (+4.68%) (*p* = 0.049).

**Figure 2:**
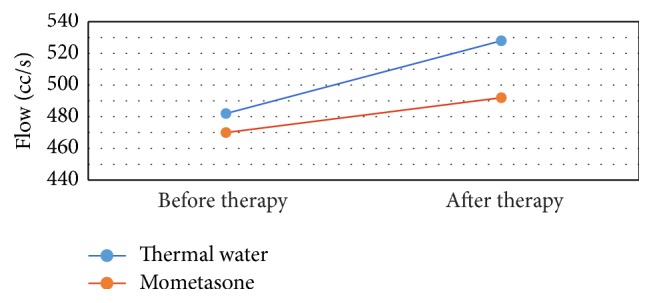
The patients (54) treated with thermal therapy show an increase of the flow, from 482 to 528 cc/s (+9.54%). In the group treated with mometasone, the flow switches from 470 to 492 cc/s (+4.68%).

**Figure 3:**
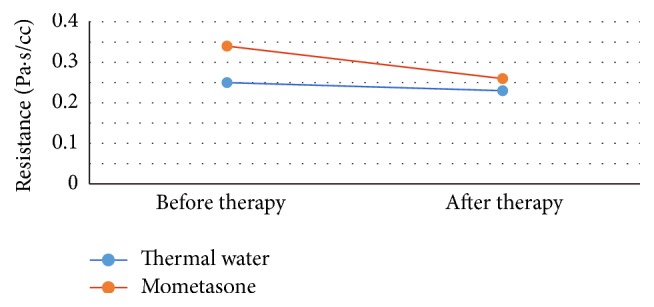
The resistance results decreased in the group treated with thermal waters, switching from a mean of 0.25 to 0.23 Pa·s/cc. In the group treated with mometasone the mean of resistance switches from 0.34 Pa·s/cc before treatment to 0.26 Pa·s/cc.

**Figure 4:**
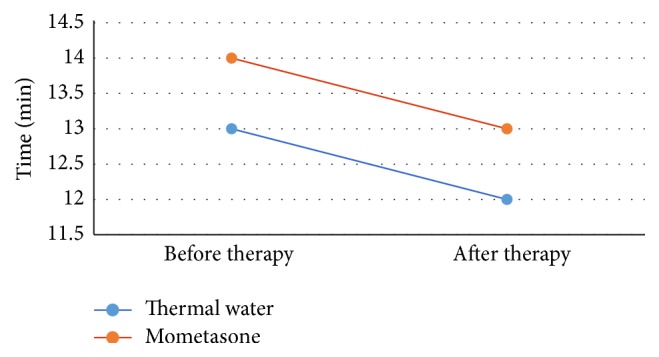
The figure shows the variance of mucociliary transport time, direct indication of mucociliary clearance function. The mean switches from 13 to 12 minutes in the group which has followed the thermal therapy and from 14 to 13 minutes in the group treated with mometasone.

Resistance decreased from a mean of 0.25 Pa/cc to 0.23 Pa/cc (−8.0%) (*p* = 0.76) in the thermal treated group and from a mean of 0.34 Pa/cc to 0.26 Pa/cc (−23.5%) (*p* = 0.093) in the control group. Mucociliary transport time improved significantly in the thermal treated group (*p* = 0.0000012) and in the mometasone treated group (*p* = 0.00324). Mean MCT time fell from 13 to 12 min in group A and from 14 to 13 min in controls (Table 4 and Figures 2, 3, and 4).

**Figure 5:**
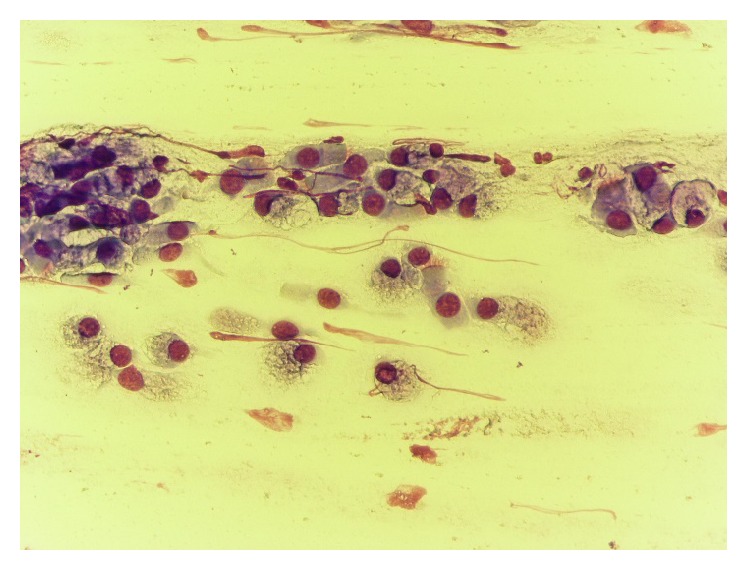
Large amount of goblet cells, before treatment.

**Figure 6:**
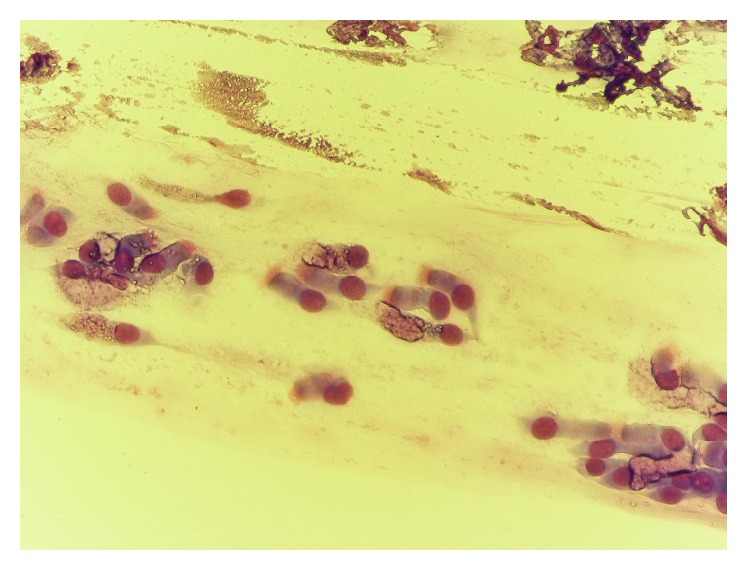
Normal rhinocytogram. Prevalence of ciliated cells (after treatment).

**Figure 7:**
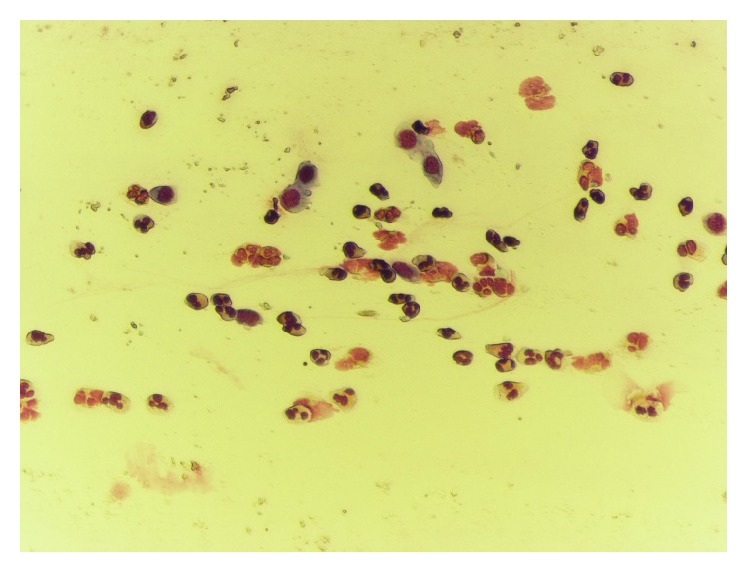
Neutrophil rhinopathy, before treatment.

**Figure 8:**
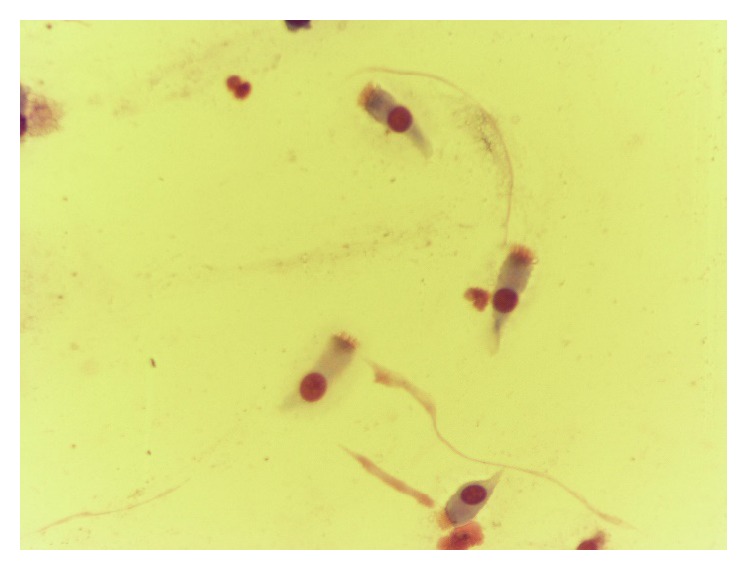
Prevalence of ciliated cells, after treatment.

**Figure 9:**
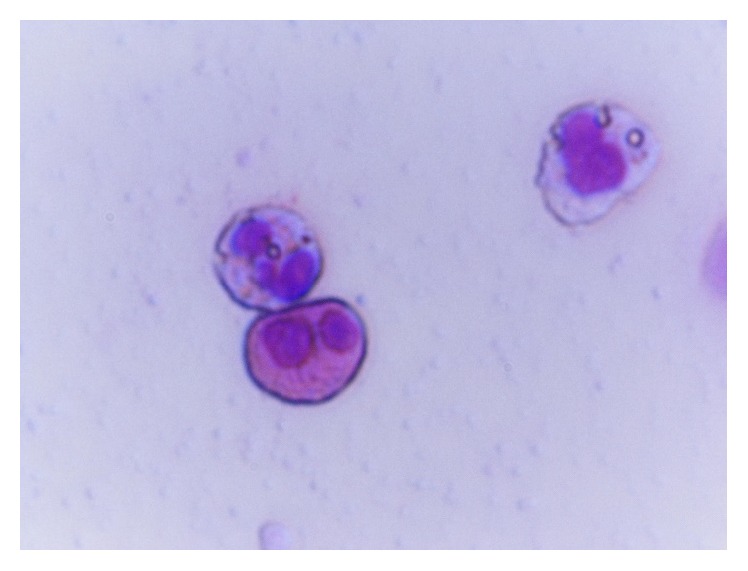
Neutrophil and eosinophil rhinopathy, before treatment.

**Figure 10:**
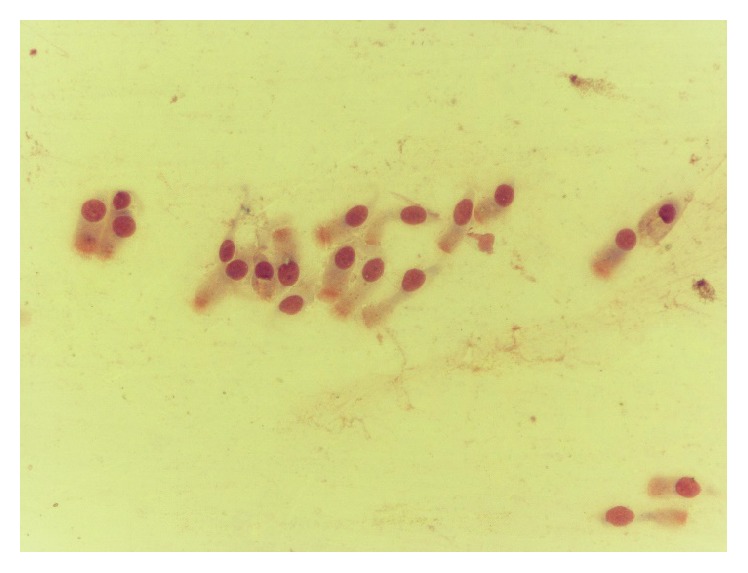
Normal rhinocytogram, after treatment.

**Figure 11:**
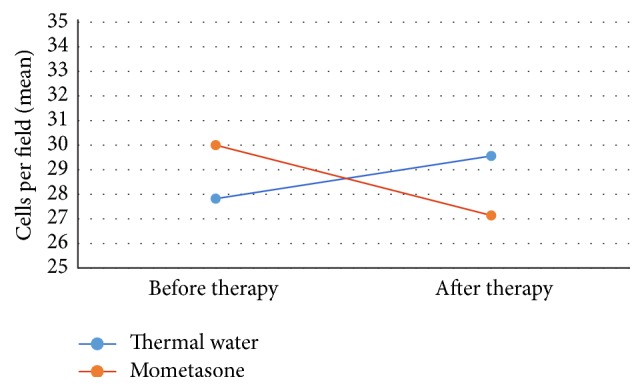
After thermal water therapy, the amount of goblet cells remains steady; it is decreased in the patients who followed mometasone therapy (from 30 to 24.4 cells per field).

**Figure 12:**
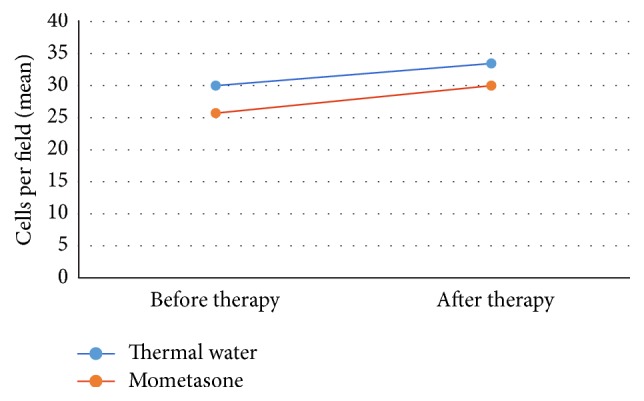
The patients treated with thermal water showed an increase of ciliated cells, from a mean of 30 to a mean of 33.47 cells per field. The patients treated with mometasone show a ciliated cell amount increase as well (from 25.7 to 30 cells per field).

With regard to cytological analysis, an increase in ciliated cells from a mean of 30 to 33.47 per microscope field was recorded in thermal treated patients (46 cases) as well as a decrease in neutrophils from a mean of 8 to 4 per field and in eosinophils from 0.26 to 0.065 per field. Patients treated with mometasone showed an increase in ciliated cells from 25.7 to 30 per field and a decrease in muciparous goblet cells from 30 to 27.4 per field (Table 5 and Figures 5, 6, 7, 8, 9, 10, 11, and 12).

No difference was recorded in the outcomes between mono- and polysensitized patients.

The cycle of thermal inhalations was well tolerated and no side effects were reported by patients. Among patients treated with mometasone furoate nasal spray, two reported annoying pruritus and three complained of moderate epistaxis.

## 4. Discussion

Nasal irrigation using saline solutions has been recommended as complementary treatment of AR in several studies and international guidelines [13–18]. Its efficacy has been clearly established in a systematic review with meta-analysis [19]: ten prospective randomized controlled studies with a total of 400 patients were considered in the review. Meta-analysis was performed with regard to the parameters “nasal symptom score,” “medicine consumption,” “mucociliary clearance time,” and “quality of life” in terms of the respective absolute improvement in comparison between the beginning and the end of the study. The review showed that nasal irrigation with saline solution in AR results in the improvement of symptoms, quality of life, and MCT; thus it is effective on subjective and objective parameters and in children, adolescents, and adults including pregnant women. However the heterogeneity of the analyzed studies regarding type, amount, and timing of nasal irrigation and the use of different saline solutions (isotonic, hypertonic) asks for additional studies to be performed in the future to clarify the questions as to the optimal salt concentration and mode of application.

A systematic review with meta-analysis [20] on the effectiveness of thermal water treatment in upper respiratory tract diseases has been recently published. 13 studies were included in the meta-analysis, 7 of which were randomized and controlled. Isotonic sodium chloride solution was used for control groups and drinking water or distilled water for the placebo groups. Definitively the review states that thermal waters have not only a function of cleansing, massage, and dilution, but fully therapeutic function as well demonstrating clear advantages on objective parameters, such as MCT, nasal flow, nasal resistance, and IgE concentration, over isotonic saline solution and placebo.

The effect of antihistamine medications on ciliary function and nasal patency has been tested in literature as well. Loratadine, levocabastine, and xylometazoline have not showed an enhancement of mucociliary function in terms of mucociliary transport time reduction and ciliary beat frequency [21–23]. Azelastine seems to cause a reduction of ciliary activity [24].

Nasal airways resistance and mucous secretions are reduced by antihistamine therapy [25, 26].

At present, there is a lack of studies which compare the traditional therapies with thermal therapy, following objective parameters.

Our study showed that inhalation treatment with radioactive hydrofluoric thermal water for two weeks produces an objective clinical and cytological improvement in allergic patients, similar to that obtained with mometasone furoate nasal spray. Specifically, inhalation of thermal waters brought about an improvement in bilateral nasal flow and hence a decrease in resistance to air flow. Mucociliary function was also modified by thermal therapy: clearance time decreased similar to the decrease obtained with mometasone therapy.

Cytological examination showed effects of thermal therapy over different cell populations. In particular, it proved effective in restoring correct mucociliary function, since it led to an increase in ciliated cells and a stabilisation of goblet cells parallel to the mucociliary clearance improvement. Thermal therapy also contributed to alleviation of chronic inflammation, since the neutrophils and eosinophils population decreased after treatment.

The SNOT score decreased both after water thermal therapy and after mometasone, showing the benefits of thermal treatment in the perception of allergic rhinitis symptoms.

## Figures and Tables

**Table 1:** Physical-chemical features of Merano thermal water.

Parameters	Results	Unit of measurement
Atmospheric pressure	981	mbar
Air temperature	22	°C
Temperature at source	23.4	°C
Colour	Colourless	
Odour	Odourless	
Savour	Normal	
Deposit	Absent	
Acidity (pH)	7.48	
Conductivity (at source)	77	*μ*S/cm
Total hardness	3.2	°F
Alkalinity (as CO_3_^−^)	0	mg/L
Alkalinity (as HCO_3_^−^)	34	mg/L
Oxidability	<0.5	mg/L
Ammonium ion (NH_4_^+^)	<0.02	mg/L
Nitrites (NO_2_^−^)	<0.002	mg/L
Fluoride (F^−^)	1.3	mg/L
Chloride (Cl^−^)	<1.0	mg/L
Nitrate (NO_3_^−^)	0.6	mg/L
Sulphate (SO_4_^−^)	9	mg/L
Radon concentration	246	Bq/L

**Table 2:** CONSORT diagram showing the allocation of patients during eligibility, enrolment, and follow-up phases.

Randomization	90 eligible patients	
	Group A Thermal water	Group B Mometasone
Enrolment	54	36
*Drop-outs*	*8 patients did not attend the follow-up*	*None*
Present at follow-up	46	36
*Drop-outs*	*5 patients: resistance and mucociliary transport were impossible to assess*	*None*
Completed a full valid follow-up	41	36

**Table 3:** The table shows the statistical analysis of SNOT scores according to Wilcoxon test, expressed in absolute values. In the patients treated with thermal water, the mean switches from 29 points before treatment to 20 points after treatment. In those patients who underwent mometasone spray therapy, the mean decreases as well, from 38 to 22. *p* value is below 0,05 in both groups.

	Before therapy	After therapy	*p*
SNOT score of thermal water	20/29/36	16/20/26	0,00000344
SNOT score of mometasone	29/38/45	15/22/29	0,0000211

**Table 4:** The table shows the results of statistical analysis according to Wilcoxon test, comparing the flow and resistance data got by active anterior rhinomanometry and the mucociliary time data, before and after thermal water therapyand mometasone nasal spray. In 46 cases encompassed by the study with *thermal water therapy (w)*, we notice increase of combined flow (right + left) after thermal therapy, switching the mean from 482 cc/s before therapy to 528 cc/s after therapy (*p* = 0,168). The data refers to first quartile/mean/third quartile. After the therapy with *mometasone nasal spray (m)* (36 cases), an increase of flow (*dx* + *sx*) is noticed, switching the mean from 470 cc/s to 492 cc/s with *p* = 0,049; the value of resistance, from 0,34, reduces to 0,26 Pa/cc with *p* = 0,093. Mucociliary transport time decreases from 14 minutes to 13 with *p* = 0,00324. The data refers to first quartile/mean/third quartile.

		Before therapy	After therapy	*p*
Flow (cc/s)	Left (*N* = 46) w	176/234/340	180/250/436	0,041
	Left (*N* = 36) m	110/188/232	190/268/460	0,078
	Right (*N* = 46) w	152/246/332	116/234/376	0,796
	Right (*N* = 36) m	114/220/388	228/240/296	0,495
	Total (*N* = 46) w	340/482/676	328/528/756	0,168
	Total (*N* = 36) m	352/470/604	474/492/602	0,049

Resistance (Pa/cc)	Left (*N* = 41) w	0,385/0,55/0,765	0,335/0,46/0,815	0,334
	Left (*N* = 36) m	0,56/0,68/0,965	0,32/0,5/0,77	0,142
	Right (*N* = 41) w	0,3975/0,55/0,8625	0,3675/0,48/0,8025	0,721
	Right (*N* = 36) m	0,34/0,62/0,87	0,40/0,55/0,65	0,151
	Total (*N* = 41) w	0,1975/0,25/0,405	0,17/0,23/0,44	0,76
	Total (*N* = 36) m	0,20/0,34/0,42	0,22/0,26/0,29	0,093

Mucociliary transport (minutes)	Thermal water (*N* = 41)	12,375/13/14	11/12/12,25	0,000001208
	Mometasone (*N* = 36)	13,0/14,0/15,0	12,0/13,0/14,0	0,00324

**Table 5:** The table compares the results of cytological analysis according to Wilcoxon test in the patients who followed thermal water therapy and mometasone nasal spray. Regarding the *thermal water therapy* group, the sample of patients allowed getting significant data about ciliated cells, goblet cells, neutrophils, and eosinophils. The mean of each type of cell per microscopic field is shown, considering the whole group of 46 patients. Ciliated cells increased, from a mean of 30 to 33.47; goblet cells (considering also the standard deviation, here not reported) remain stable (from 28 to 29,5); neutrophils almost halve (*p* = 0,187); eosinophils decrease from a mean of 0,26 to 0.065 (*p* = 0,08). Regarding the cytological exam of 36 patients treated by *mometasone* there were significant outcomes concerning ciliated cells and goblet cells. In particular, the mean of ciliated cells per field increased from 25,71 to 30; the mean of goblet cells decreased from 30 to 27,14.

	Before therapy		After therapy		*p*	
	Th. water	Mom.	Th. water	Mom.	Th. water	Mom.
Ciliated cells	30	25,71	33.47	30	0,421	0,536
Goblet cells	27,82	30	29,5652	27,14	0,484	0,808
Neutrophils	8,13	6,28	4,78	6,28	0,187	1
Eosinophils	0,26	0,071	0,065	0,071	0,08	1
Basophiles	0	0	0	0		
Mast cells	0,021	0	0	0	1	
Macrophages	0	0	0,21	0,07	1	1
Lymphocytes	0	0	0	0		
Bacteria	0	0	0	0		
Fungi	0	0,14	0	0		1
